# Curvature Invariants of Statistical Submanifolds in Kenmotsu Statistical Manifolds of Constant *ϕ*-Sectional Curvature

**DOI:** 10.3390/e20070529

**Published:** 2018-07-14

**Authors:** Simona Decu, Stefan Haesen, Leopold Verstraelen, Gabriel-Eduard Vîlcu

**Affiliations:** 1 Department of Applied Mathematics, Bucharest University of Economic Studies, Piaţa Romană 6, 010374 Bucharest, Romania; 2 Romanian Academy, *Costin C. Kiriţescu* National Institute of Economic Researches—Mountain Economy Centre (CE-MONT), Calea 13 Septembrie 13, 030508 Bucharest, Romania; 3 Department of Mathematics, University of Hasselt, BE 3590 Diepenbeek, Belgium; 4 Department of Teacher Education, Thomas More University College, 2290 Vorselaar, Belgium; 5 Department of Mathematics, Katholieke Universiteit Leuven, Celestijnenlaan 200B, Box 2400, BE-3001 Leuven, Belgium; 6 Faculty of Mathematics and Computer Science, Research Center in Geometry, Topology and Algebra, University of Bucharest, Str. Academiei 14, Sector 1, 70109 Bucharest, Romania; 7 Department of Cybernetics, Economic Informatics, Finance and Accountancy, Petroleum-Gas University of Ploieşti, Bd. Bucureşti 39, 100680 Ploieşti, Romania

**Keywords:** casorati curvature, statistical submanifold, Kenmotsu statistical manifold, dual connections

## Abstract

In this article, we consider statistical submanifolds of Kenmotsu statistical manifolds of constant ϕ-sectional curvature. For such submanifold, we investigate curvature properties. We establish some inequalities involving the normalized δ-Casorati curvatures (extrinsic invariants) and the scalar curvature (intrinsic invariant). Moreover, we prove that the equality cases of the inequalities hold if and only if the imbedding curvature tensors *h* and $h^{*}$ of the submanifold (associated with the dual connections) satisfy $h=-h^{*}$, i.e., the submanifold is totally geodesic with respect to the Levi–Civita connection.

## 1. Introduction

A fundamental problem in the general theory of Riemannian submanifolds is to establish simple relationships between the main *intrinsic invariants* and the main *extrinsic invariants* of the submanifolds [1]. Obviously, such simple relationships can be provided by certain types of inequalities. Furthermore, the investigation of *ideal submanifolds* in a space form, namely the study of submanifolds satisfying the equality case of such inequalities, is another basic problem of this field [2].

On one hand, it is well known that the theory of Chen invariants provides solutions to these problems. Chen proved initially in [1] some optimal inequalities between the *intrinsic δ-curvatures of Chen* and the *extrinsic squared mean curvature* of submanifolds in a real space form. Later, these sharp inequalities (called *Chen inequalities*) have been extended for different types of submanifolds in various ambient spaces, for example, arbitrary Riemannian manifolds, Kähler manifolds, and Kenmotsu space forms (see [3] and the references therein). The *Chen ideal submanifolds* have also been investigated, i.e., the submanifolds that do realize an optimal equality in Chen inequalities (see, e.g., the recent work [4]).

On the other hand, the study of *δ-Casorati curvatures*, initiated in [5,6], offers new solutions to the above problems. The Casorati curvature is a concept preferred by Casorati over the traditional Gauss curvature and the mean curvature because it corresponds better with the common intuition of curvature [7]. Koenderink [8] and Verstraelen [9] studied the meaning of the Casorati curvature in geometry and other fields, like human/computer vision. Notice that some results in terms of isotropical Casorati curvature of production surfaces were obtained in [10]. A geometrical interpretation of the Casorati curvature of submanifolds in Riemannian manifolds was given in [11]. Very recently, Brubaker et al. [12] gave a geometric interpretation of Cauchy–Schwarz inequality in terms of Casorati curvature. The first optimal inequalities involving the *extrinsic δ-Casorati curvatures* and the *intrinsic scalar curvature* of submanifolds in real space forms were proved in [5,6]. Later, this knowledge has been extended (e.g., see [13,14,15,16,17,18,19]). Submanifolds for which these equalities hold are called *Casorati ideal submanifolds*. Recently, Lee et al. [20] studied optimal inequalities in terms of δ-Casorati curvatures of submanifolds in Kenmotsu space forms. We recall that the *Kenmotsu geometry* is an area of contact geometry initiated by Kenmotsu in [21], with many applications, e.g., in physics (geometrical optics, classical mechanics, thermodynamics, geometric quantization) and control theory [22]. This geometry arose in a natural way in the paper [21], where the author proposed to investigate the geometric properties of the warped product of the complex space with the real line. It is indeed a natural problem since this product is one of the three classes in Tanno’s classification of connected almost contact Riemannian manifolds with an automorphism group of maximum dimension [22].

In 1985, Amari defined the notions of *statistical manifold* and *conjugate connection* in the basic study on information geometry [23]. It is well known that there is a deep relationship between statistical manifolds and entropy [24]. We notice that a characterization of the class of statistical manifolds having coordinate systems for which the relative entropy (Kullback–Leibler divergence) is a Bregman divergence was obtained by Nagaoka [25]. On the other hand, Dillen et al. studied in [26] the conjugate (dual) connections on a semi-Riemannian manifold. Moreover, the authors found in [26] a new formulation of Radon’s theorem in affine differential geometry, proving a necessary and sufficient condition for the existence of an affine immersion which realizes the induced affine connection and the induced affine second fundamental form. The geometry of statistical manifolds also provides interesting issues for differential geometry, statistics, machine learning, etc. (see, e.g., [27,28,29,30,31,32]). In particular, the differential geometry field is focused on topics such as submanifold theory of statistical manifolds [33], Hessian geometry [34], statistical submersions [35], complex manifold theory of statistical manifolds ([29,36,37]), contact theory on statistical manifolds [38], and quaternionic theory on statistical manifolds [39]. For the above problems, Aydin et al. obtained Chen–Ricci inequalities [40] and a generalized Wintgen inequality [41] for submanifolds in statistical manifolds of constant curvature. Moreover, Lee et al. established optimal inequalities involving the Casorati curvatures and the normalized scalar curvature on submanifolds of statistical manifolds of constant curvature [42]. These inequalities were extended by Aquib and Shahid [43] in the setting of statistical submanifolds in quaternion Kähler-like statistical space forms. On the other hand, Mihai et al. [44] proved an Euler inequality and a Chen–Ricci inequality for statistical submanifolds of Hessian manifolds of constant Hessian curvature.

Recently, Furuhata et al. [45] introduced the concept of *Kenmotsu statistical manifold*, which is locally obtained as the warped product of a holomorphic statistical manifold and a line. They proved that a Kenmotsu statistical manifold of constant ϕ-sectional curvature is constructed from a special Kähler manifold, which is an important example of holomorphic statistical manifold. In this respect, the authors considered the warped product of two statistical manifolds and investigated the statistical sectional curvature of this warped product. Then, they equipped a Kenmotsu manifold with a natural affine connection and studied how to construct a Kenmotsu statistical manifold of constant ϕ-sectional curvature as the warped product of a holomorphic statistical manifold and a line. It would thus be of interest to study inequalities concerning the extrinsic δ-Casorati curvatures for statistical submanifolds in Kenmotsu statistical manifolds. In this article, we establish inequalities in terms of the extrinsic normalized δ-Casorati curvatures and the intrinsic scalar curvature of statistical submanifolds in Kenmotsu statistical manifolds of constant ϕ-sectional curvature. The methodology is based on a constrained extremum problem. Furthermore, we study the equality cases. We derive that the equality at all points characterizes the submanifolds that are totally geodesic with respect to the Levi–Civita connection.

## 2. Preliminaries

Let $(\bar{M}$, $\bar{g}$) be a (2n+1)-dimensional Riemannian manifold with an affine connection $\bar{\nabla }$. Denote by Γ(E) the set of sections of a vector bundle $E\to \bar{M}$, e.g., $\Gamma (T{\bar{M}}^{\left(p,q\right)})$ means the set of all tensor fields on $\bar{M}$ of type (p,q). Let $\bar{T}\in \Gamma \left(T{\bar{M}}^{\left(1,2\right)}\right)$ be the torsion tensor field of $\bar{\nabla }$. A pair $\left(\bar{\nabla },\bar{g}\right)$ is called a *statistical structure* [45] on $\bar{M}$ if (1) the torsion tensor field $\bar{T}$ of $\bar{\nabla }$ vanishes and (2) the Codazzi equation $\left({\bar{\nabla }}_{X}\bar{g}\right)\left(Y,Z\right)=\left({\bar{\nabla }}_{Y}\bar{g}\right)\left(X,Z\right)$ holds for any *X*, *Y*, *Z* $\in \Gamma (T\bar{M})$. Any connection $\bar{\nabla }$ satisfying the condition ${\bar{\nabla }}_{X}Y-{\bar{\nabla }}_{Y}X=XY-YX$ for *X*, *Y* $\in \Gamma (T\bar{M})$ is called *torsion-free*.

A *statistical manifold* [33] is a Riemannian manifold $(\bar{M}$, $\bar{g})$ in which there exists a pair of torsion-free affine connections $\bar{\nabla }$ and ${\bar{\nabla }}^{*}$ that satisfy

$$Z\;\bar{g}\left(X,Y\right)=\bar{g}\left({\bar{\nabla }}_{Z}X,Y\right)+\bar{g}\left(X,{\bar{\nabla }}_{Z}^{*}Y\right),$$

for any *X*, *Y*, *Z* $\in \Gamma (T\bar{M})$. The statistical manifold is denoted by the triplet $(\bar{M}$, $\bar{g}$, $\bar{\nabla })$. The connections $\bar{\nabla }$ and ${\bar{\nabla }}^{*}$ are called *dual connections*.

**Remark** **1.** *If $(\bar{M}$, $\bar{g}$, $\bar{\nabla })$ is a statistical manifold, then we notice as follows:*

*1.*  *${\left({\bar{\nabla }}^{*}\right)}^{*}=\bar{\nabla }$.*
*2.*  *$(\bar{M}$, $\bar{g}$, ${\bar{\nabla }}^{*})$ is also a statistical manifold.*
*3.*  *Any torsion-free affine connection $\bar{\nabla }$ always has a dual connection ${\bar{\nabla }}^{*}$ performing*
  (1) $$\bar{\nabla }+{\bar{\nabla }}^{*}=2{\bar{\nabla }}^{0},$$
  *where ${\bar{\nabla }}^{0}$ denotes the Levi–Civita connection on $\bar{M}$.*

Let *M* be an (m+1)-dimensional submanifold of a (2n+1)-dimensional statistical manifold $\left(\bar{M},\bar{g}\right)$ and *g* the induced metric on *M*. Then, the Gauss formulas are as follows:

$$\begin{array}{c} {\bar{\nabla }}_{X}Y=\nabla_{X}Y+h\left(X,Y\right),\\ {\bar{\nabla }}_{X}^{*}Y=\nabla_{X}^{*}Y+h^{*}\left(X,Y\right), \end{array}$$

for any X,Y∈Γ(TM), where *h* and $h^{*}$ are symmetric (0,2)-tensors, called the *imbedding curvature tensor of M in $\bar{M}$ for $\bar{\nabla }$*, and the *imbedding curvature tensor of M in $\bar{M}$ for ${\bar{\nabla }}^{*}$*, respectively.

Denote by *R* and $\bar{R}$ the curvature tensors fields associated with ∇ and $\bar{\nabla }$, respectively. Then the *Gauss equation* for the submanifold *M* of $\bar{M}$ (with respect to the connection $\bar{\nabla }$) is [33]

(2) $$\begin{array}{c} \bar{g}\left(\bar{R}\left(X,Y\right)Z,W\right)=g\left(R\left(X,Y\right)Z,W\right)+\bar{g}\left(h\left(X,Z\right),h^{*}\left(Y,W\right)\right)-\bar{g}\left(h^{*}\left(X,W\right),h\left(Y,Z\right)\right), \end{array}$$

for any X,Y,Z,W∈Γ(TM).

Similarly, if $R^{*}$ and ${\bar{R}}^{*}$ denote the curvature tensors fields associated with the connections $\nabla^{*}$ and ${\bar{\nabla }}^{*}$, respectively, then the *Gauss equation* with respect to the connection ${\bar{\nabla }}^{*}$ is [33]

(3) $$\begin{array}{c} \bar{g}\left({\bar{R}}^{*}\left(X,Y\right)Z,W\right)=g\left(R^{*}\left(X,Y\right)Z,W\right)+\bar{g}\left(h^{*}\left(X,Z\right),h\left(Y,W\right)\right)-\bar{g}\left(h\left(X,W\right),h^{*}\left(Y,Z\right)\right), \end{array}$$

for any X,Y,Z,W∈Γ(TM).

If ($\bar{M}$, $\bar{g}$, $\bar{\nabla })$ is a statistical manifold and *M* is a submanifold of $\bar{M}$, then (M,g,∇) is also a statistical manifold with the induced connection ∇ and the induced metric *g*.

For a statistical manifold (M,g,∇), the tensor field $S\in \Gamma (TM^{\left(1,3\right)})$ called the *statistical curvature tensor field of* (M,g,∇) is defined as [46]:

(4) $$S\left(X,Y\right)Z=\frac{1}{2}\left\{R\left(X,Y\right)Z+R^{*}\left(X,Y\right)Z\right\},$$

for X,Y,Z∈Γ(TM).

For a statistical structure $\left(\bar{g},\bar{\nabla }\right)$ on $\bar{M}$, we set $\bar{K}=\bar{\nabla }-{\bar{\nabla }}^{0}$ according to [45], which implies:

(5) $$\begin{array}{c} \bar{\nabla }={\bar{\nabla }}^{0}+\bar{K}. \end{array}$$

It follows that the tensor field $\bar{K}\in \Gamma \left(T{\bar{M}}^{\left(1,2\right)}\right)$ satisfies

$${\bar{K}}_{X}Y={\bar{K}}_{Y}X,\;g\left({\bar{K}}_{X}Y,Z\right)=g\left(Y,{\bar{K}}_{X}Z\right),$$

for any $X,Y,Z\in \Gamma (T\bar{M})$. We denote

$${\bar{K}}_{X}Y=\bar{K}\left(X,Y\right).$$

Let $\pi =span_{R}\left\{v,w\right\}$ be a two-dimensional subspace of $T_{p}M$, for p∈M. The *sectional curvature* of *M* for π is defined by [46]:

(6) $$\begin{array}{c} \sigma \left(\pi \right)=\frac{g(S(v,w)w,v)}{g\left(v,v\right)g\left(w,w\right)-g^{2}\left(v,w\right)}. \end{array}$$

Let $\left\{e_{1},\ldots ,e_{m+1}\right\}$ be an orthonormal basis of the tangent space $T_{p}M$, for p∈M, and let $\left\{e_{m+2},\ldots ,e_{2n+1}\right\}$ be an orthonormal basis of the normal space $T_{p}^{⊥}M$. The *scalar curvature* τ at *p* is given by

(7) $$\begin{array}{c} \tau \left(p\right)=\sum_{1\leq i<j\leq m+1}\sigma \left(e_{i}\wedge e_{j}\right)=\sum_{1\leq i<j\leq m+1}g\left(S\left(e_{i},e_{j}\right)e_{j},e_{i}\right), \end{array}$$

and the *normalized scalar curvature* ρ of *M* is defined as

(8) $$\rho =\frac{2\tau }{n(n-1)}.$$

The *mean curvature* vector fields of *M*, denoted by *H* and $H^{*}$, are given by:

$$H=\frac{1}{m+1}\sum_{i=1}^{m+1}h\left(e_{i},e_{i}\right),\;\;H^{*}=\frac{1}{m+1}\sum_{i=1}^{m+1}h^{*}\left(e_{i},e_{i}\right).$$

We remark that, from Equation (1), we derive $2h^{0}=h+h^{*}$ and therefore $2H^{0}=H+H^{*}$, where $h^{0}$ and $H^{0}$ are the second fundamental form and the mean curvature field of *M*, respectively, with respect to the Levi–Civita connection $\nabla^{0}$ on *M*.

Then, the *squared mean curvatures* of the submanifold *M* in $\bar{M}$ are calculated by:

$${∥H∥}^{2}=\frac{1}{{\left(m+1\right)}^{2}}\sum_{\alpha =m+2}^{2n+1}{\left(\sum_{i=1}^{m+1}h_{ii}^{\alpha }\right)}^{2},\;\;{∥H^{*}∥}^{2}=\frac{1}{{\left(m+1\right)}^{2}}\sum_{\alpha =m+2}^{2n+1}{\left(\sum_{i=1}^{m+1}h_{ii}^{*\alpha }\right)}^{2},$$

where $h_{ij}^{\alpha }=\bar{g}\left(h\left(e_{i},e_{j}\right),e_{\alpha }\right)$ and $h_{ij}^{*\alpha }=\bar{g}\left(h^{*}\left(e_{i},e_{j}\right),e_{\alpha }\right)$, for i,j∈{1,…,m+1}, α∈{m+2,…,2n+1}.

The *Casorati curvatures* of the submanifold *M* in $\bar{M}$ are defined by the squared norms of *h* and $h^{*}$ over the dimension (m+1), denoted by C and $C^{*}$, respectively, as follows:

$$C=\frac{1}{m+1}{∥h∥}^{2}=\frac{1}{m+1}\sum_{\alpha =m+2}^{2n+1}\sum_{i,j=1}^{m+1}{\left(h_{ij}^{\alpha }\right)}^{2},$$

$$C^{*}=\frac{1}{m+1}{∥h^{*}∥}^{2}=\frac{1}{m+1}\sum_{\alpha =m+2}^{2n+1}\sum_{i,j=1}^{m+1}{\left(h_{ij}^{*\alpha }\right)}^{2}.$$

Let *L* be an *s*-dimensional subspace of $T_{p}M$, s≥2 and let $\left\{e_{1},\ldots ,e_{s}\right\}$ be an orthonormal basis of *L*. Hence, the Casorati curvatures C(L) and $C^{*}\left(L\right)$ of *L* are given by:

$$C\left(L\right)=\frac{1}{s}\sum_{\alpha =m+2}^{2n+1}\sum_{i,j=1}^{s}{\left(h_{ij}^{\alpha }\right)}^{2},\;\;C^{*}\left(L\right)=\frac{1}{s}\sum_{\alpha =m+2}^{2n+1}\sum_{i,j=1}^{s}{\left(h_{ij}^{*\alpha }\right)}^{2}.$$

The *normalized δ-Casorati curvatures* $\delta_{C}\left(m\right)$ and ${\hat{\delta }}_{C}\left(m\right)$ of the submanifold $M^{n}$ are given by

$$\delta_{C}{\left(m\right)|}_{p}=\frac{1}{2}C{|}_{p}+\frac{m+2}{2(m+1)}\inf\left\{C\left(L\right)|L\;a\;\mathrm{hyperplane}\;\mathrm{of}\;T_{p}M\right\}$$

and

$${\hat{\delta }}_{C}{\left(m\right)|}_{p}=2C{|}_{p}-\frac{2m+1}{2(m+1)}\sup\left\{C\left(L\right)|L\;a\;\mathrm{hyperplane}\;\mathrm{of}\;T_{p}M\right\}.$$

Similarly, we can define the *dual normalized $\delta^{*}$-Casorati curvatures* $\delta_{C}^{*}\left(m\right)$ and ${\hat{\delta }}_{C}^{*}\left(m\right)$ of the submanifold *M* in $\bar{M}$, just replacing C by $C^{*}$ in the last two relations:

$$\delta_{C}^{*}{\left(m\right)|}_{p}=\frac{1}{2}C^{*}{|}_{p}+\frac{m+2}{2(m+1)}\inf\left\{C^{*}\left(L\right)|L\;a\;\mathrm{hyperplane}\;\mathrm{of}\;T_{p}M\right\}$$

and

$${\hat{\delta }}_{C}^{*}{\left(m\right)|}_{p}=2C^{*}{|}_{p}-\frac{2m+1}{2(m+1)}\sup\left\{C^{*}\left(L\right)|L\;a\;\mathrm{hyperplane}\;\mathrm{of}\;T_{p}M\right\}.$$

The *generalized normalized δ-Casorati curvatures* $\delta_{C}\left(r;m\right)$ and ${\hat{\delta }}_{C}\left(r;m\right)$ of *M* in $\bar{M}$ are defined in [6] as:

$$\delta_{C}{\left(r;m\right)|}_{p}=r\;C{|}_{p}+a\left(r\right)\;\inf\left\{C\left(L\right)|L\;a\;\mathrm{hyperplane}\;\mathrm{of}\;T_{p}M\right\},$$

if 0<r<m(m+1), and

$${\hat{\delta }}_{C}{\left(r;m\right)|}_{p}=r\;C{|}_{p}+a\left(r\right)\;\sup\left\{C\left(L\right)|L\;a\;\mathrm{hyperplane}\;\mathrm{of}\;T_{p}M\right\},$$

if r>m(m+1), whereby a(r) is set as

$$\begin{array}{c} a\left(r\right)=\frac{m\left(r+m+1\right)\left(m^{2}+m-r\right)}{(m+1)r}, \end{array}$$

for any positive real number *r*, different from m(m+1).

Moreover, the *dual generalized normalized $\delta^{*}$-Casorati curvatures* $\delta_{C}^{*}\left(r;m\right)$ and ${\hat{\delta^{*}}}_{C}\left(r;m\right)$ of the submanifold *M* in $\bar{M}$ are given by:

$$\delta_{C}^{*}{\left(r;m\right)|}_{p}=r\;C^{*}{|}_{p}+a\left(r\right)\;\inf\left\{C^{*}\left(L\right)|L\;a\;\mathrm{hyperplane}\;\mathrm{of}\;T_{p}M\right\},$$

if 0<r<m(m+1), and

$${\hat{\delta }}_{C}^{*}{\left(r;m\right)|}_{p}=r\;C^{*}{|}_{p}+a\left(r\right)\;\sup\left\{C^{*}\left(L\right)|L\;a\;\mathrm{hyperplane}\;\mathrm{of}\;T_{p}M\right\},$$

if r>m(m+1), whereby a(r) is set above.

Obviously, the (dual) generalized normalized δ- and $\delta^{*}$-Casorati curvatures are a natural generalization of the (dual) normalized δ- and $\delta^{*}$-Casorati curvatures, due the fact that $\delta_{C}\left(m\right)$, ${\hat{\delta }}_{C}\left(m\right)$, $\delta_{C}^{*}\left(m\right)$ and ${\hat{\delta }}_{C}^{*}\left(m\right)$ can be recovered from $\delta_{C}\left(r;m\right)$, ${\hat{\delta }}_{C}\left(r;m\right)$, $\delta_{C}^{*}\left(r;m\right)$ and ${\hat{\delta^{*}}}_{C}\left(r;m\right),$ respectively, for some particular values of *r* as follows:

(9) $$\delta_{C}\left(m\right)=\frac{1}{m(m+1)}\delta_{C}\left(\frac{m(m+1)}{2};m\right),\;{\hat{\delta }}_{C}\left(m\right)=\frac{1}{m(m+1)}{\hat{\delta }}_{C}\left(2m(m+1);m\right),$$

(10) $$\delta_{C}^{*}\left(m\right)=\frac{1}{m(m+1)}\delta_{C}^{*}\left(\frac{m(m+1)}{2};m\right),\;{\hat{\delta^{*}}}_{C}\left(m\right)=\frac{1}{m(m+1)}{\hat{\delta^{*}}}_{C}\left(2m(m+1);m\right).$$

We recall that a statistical submanifold (M,g,∇) in $\left(\bar{M},\bar{g},\bar{\nabla }\right)$ is called *totally geodesic* with respect to the connection $\bar{\nabla }$ if the second fundamental form *h* of *M* for $\bar{\nabla }$ vanishes identically [46].

Consider that $\left(\bar{M},\bar{g}\right)$ is a (2n+1)-dimensional *almost contact metric manifold* with the structure tensors ($\bar{g},\phi ,\xi$), where $\bar{g}\in \Gamma \left(T{\bar{M}}^{\left(0,2\right)}\right)$ is the Riemannian metric on $\bar{M}$, $\phi \in \Gamma (T{\bar{M}}^{\left(1,1\right)})$, $\xi \in \Gamma (T\bar{M})$. These structure tensors satisfy:

$$\phi \xi =0,\;\;\bar{g}\left(\xi ,\xi \right)=1,\;\;\phi^{2}X=-X+\eta \left(X\right)\xi ,\;\;\bar{g}\left(\phi X,Y\right)+\bar{g}\left(X,\phi Y\right)=0,$$

where η is a 1-form on $\bar{M}$ such that $\eta \left(X\right)=\bar{g}\left(X,\xi \right)$, for any $X,Y\in \Gamma (T\bar{M})$.

The almost contact metric manifold $\left(\bar{M},\bar{g}\right)$ is said to be a *Kenmotsu manifold* if the formulas:

$$\left({\bar{\nabla }}_{X}^{0}\phi \right)\left(Y\right)=\bar{g}\left(\phi X,Y\right)\xi -\eta \left(Y\right)\phi X,$$

$${\bar{\nabla }}_{X}^{0}\xi =X-\eta \left(X\right)\xi$$

hold for any $X,Y\in \Gamma (T\bar{M})$.

We outline that the Kenmotsu geometry turns out to be a valuable chapter of contact geometry with many applications in theoretical physics, providing an excellent setting to model space time near black holes or bodies with large gravitational fields [47]. One reason to study the Kenmotsu manifolds is that this class of manifolds is one of the three classes in Tanno’s classification of connected almost contact metric manifolds whose automorphism group has a maximum dimension. Another reason is that these manifolds are in some sense complementary to Sasaki manifolds: while some properties of Kenmotsu manifolds can be obtained deforming slowly properties of Sasaki manifolds, others are very different [22].

Denote ($\bar{M},\bar{g},\phi ,\xi$) a Kenmotsu manifold and let ($\bar{\nabla },\bar{g}$) be a statistical structure on $\bar{M}$, where $\bar{\nabla }={\bar{\nabla }}^{0}+\bar{K}$ is given as in Equation (5). A quadruple ($\bar{\nabla },\bar{g},\phi ,\xi$) is called a *Kenmotsu statistical structure* [45] on $\bar{M}$ if the relation:

$$\bar{K}\left(X,\phi Y\right)+\phi \bar{K}\left(X,Y\right)=0$$

holds for any $X,Y\in \Gamma (T\bar{M})$.

A manifold equipped with a Kenmotsu statistical structure is called a *Kenmotsu statistical manifold*. Notice that if ($\bar{M},\bar{\nabla },\bar{g},\phi ,\xi$) is a Kenmotsu statistical manifold, then ($\bar{M},{\bar{\nabla }}^{*},\bar{g},\phi ,\xi$) is too.

A Kenmotsu statistical manifold ($\bar{M},\bar{\nabla },\bar{g},\phi ,\xi$) is of constant ϕ-sectional curvature *c* if and only if [45]:

(11) $$\begin{array}{cc} \bar{S}\left(X,Y\right)Z & =\;\frac{c-3}{4}\left\{\bar{g}\left(Y,Z\right)X-\bar{g}\left(X,Z\right)Y\right)\}\\ & +\;\frac{c+1}{4}\{\bar{g}\left(\phi Y,Z\right)\phi X-\bar{g}\left(\phi X,Z\right)\phi Y-2\bar{g}\left(\phi X,Y\right)\phi Z\\ & -\;\bar{g}\left(Y,\xi \right)\bar{g}\left(Z,\xi \right)X+\bar{g}\left(X,\xi \right)\bar{g}\left(Z,\xi \right)Y\\ & +\;\bar{g}\left(Y,\xi \right)\bar{g}\left(Z,X\right)\xi -\bar{g}\left(X,\xi \right)\bar{g}\left(Z,Y\right)\xi \}, \end{array}$$

for any $X,Y,Z\in \Gamma (T\bar{M})$.

Let *M* be an (m+1)-dimensional statistical submanifold of a Kenmotsu statistical manifold $\left(\bar{M},\bar{\nabla },\bar{g},\phi ,\xi \right)$. Then, any vector field *X* tangent to *M* can be decomposed uniquely into its tangent and normal components PX and FX, respectively, thus:

(12) $$\begin{array}{c} \Phi X=PX+FX. \end{array}$$

Given a local orthonormal frame $\left\{e_{1},e_{2},\cdots ,e_{m+1}\right\}$ of *M*, then the squared norm of *P* is expressed by:

$${∥P∥}^{2}=\sum_{i,j=1}^{m+1}g^{2}\left(Pe_{i},e_{j}\right).$$

Next, we consider the constrained extremum problem

(13) $$\min_{x\in M}f\left(x\right).$$

The Hessian of the function *f* on the manifold $\bar{M}$ is defined by the (0,2)-tensor:

$$\mathrm{Hess}\left(f\right)\left(X,Y\right)=X\left(Y\left(f\right)\right)-\left({\bar{\nabla }}_{X}^{0}Y\right)\left(f\right),$$

where ${\bar{\nabla }}^{0}$ is the Levi–Civita connection on $\bar{M}$. We recall the following result.

**Theorem** **1.** *If the submanifold M is complete and connected, in addition to the gradient of f being normal at a point p to M, and the bilinear form $A:T_{p}M\times T_{p}M\to R$ given by:*

(14) $$A\left(X,Y\right)=\mathrm{Hess}\left(f\right)\left(X,Y\right)+\bar{g}\left(h^{0}\left(X,Y\right),\mathrm{grad}f\right),$$

*is positive definite in p, then p is an optimal solution of the problem* (13) [48].

**Remark** **2.** *If the bilinear form A defined by Equation* (14) *is positive semi-definite on the submanifold M, then the critical points of f|M, which coincide with the points where the gradient of f is normal to M, are global optimal solutions of the problem* (13) [48].

## 3. Results’ Main Inequalities

**Theorem** **2.** *Let M be an (m+1)-dimensional statistical submanifold of a (2n+1)-dimensional Kenmotsu statistical manifold ($\bar{M},\bar{\nabla },\bar{g},\phi ,\xi$) of constant ϕ-sectional curvature c. Then:*

*(i)*  *For any real number r such that 0<r<m(m+1), the generalized normalized δ-Casorati curvatures $\delta_{C}\left(r;m\right)$ and $\delta_{C}^{*}\left(r;m\right)$ satisfy*
  (15) $$\begin{array}{ccc} 2\tau & \leq & \delta_{C}^{0}\left(r;m\right)+\left(m+1\right)C^{0}-2{\left(m+1\right)}^{2}{∥H^{0}∥}^{2}\\ & + & {\left(m+1\right)}^{2}\bar{g}\left(H,H^{*}\right)+\frac{3(c+1)}{4}{∥P∥}^{2}+\frac{m}{4}\left[c\left(m-1\right)-3m-5\right], \end{array}$$
  *where $\delta_{C}^{0}\left(r,m\right)$ and $C^{0}$ are defined by $2\delta_{C}^{0}\left(r,m\right)=\delta_{C}\left(r;m\right)+\delta_{C}^{*}\left(r;m\right)$ and $2C^{0}=C+C^{*}$.*
*(ii)*  *For any real number r such that r>m(m+1), the generalized normalized δ-Casorati curvatures ${\hat{\delta }}_{C}\left(r;m\right)$ and ${\hat{\delta }}_{C}^{*}\left(r;m\right)$ satisfy*
  (16) $$\begin{array}{ccc} 2\tau & \leq & {\hat{\delta }}_{C}^{0}\left(r;m\right)+\left(m+1\right)C^{0}-2{\left(m+1\right)}^{2}{∥H^{0}∥}^{2}\\ & + & {\left(m+1\right)}^{2}\bar{g}\left(H,H^{*}\right)+\frac{3(c+1)}{4}{∥P∥}^{2}+\frac{m}{4}\left[c\left(m-1\right)-3m-5\right], \end{array}$$
  *where ${\hat{\delta }}_{C}^{0}\left(r,m\right)$ is defined by $2{\hat{\delta }}_{C}^{0}\left(r,m\right)={\hat{\delta }}_{C}\left(r;m\right)+{\hat{\delta }}_{C}^{*}\left(r;m\right).$*

*In addition, the equality cases of Equations* (15) *and* (16) *hold identically at all points p∈M if and only if the imbedding curvature tensors h and $h^{*}$ of the submanifold associated with the dual connections $\bar{\nabla }$ and ${\bar{\nabla }}^{*}$ satisfy*

(17) $$h=-h^{*}.$$

**Proof.**  From Equations (2)–(4), we get:

(18) $$\begin{array}{cc} 2\bar{g}\left(\bar{S}\left(X,Y\right)Z,W\right)= & 2g\left(S\left(X,Y\right)Z,W\right)-\bar{g}\left(h\left(Y,Z\right),h^{*}\left(X,W\right)\right)+\bar{g}\left(h\left(X,Z\right),h^{*}\left(Y,W\right)\right)\\ & -\bar{g}\left(h^{*}\left(Y,Z\right),h\left(X,W\right)\right)+\bar{g}\left(h^{*}\left(X,Z\right),h\left(Y,W\right)\right), \end{array}$$

where X,Y,Z,W∈Γ(TM).

Let $\left\{e_{1},\ldots ,e_{m+1}\right\}$ and $\left\{e_{m+2},\ldots ,e_{2n+1}\right\}$ be orthonormal bases of $T_{p}M$ and $T_{p}^{⊥}M$, respectively, for p∈M. Setting $X=Z=e_{i}$ and $Y=W=e_{j}$ for i,j∈{1,…,m+1}, and summing over 1≤i,j≤m+1 in Equation (18), we obtain:

(19) $$\begin{array}{cc} 2\tau (p)= & {\left(m+1\right)}^{2}\bar{g}\left(H,H^{*}\right)-\sum_{1\leq i,j\leq m+1}\bar{g}\left(h^{*}\left(e_{i},e_{j}\right),h\left(e_{i},e_{j}\right)\right)\\ & +\frac{3(c+1)}{4}{∥P∥}^{2}+\frac{m}{4}\left[c\left(m-1\right)-3m-5\right]. \end{array}$$

Because $2H^{0}=H+H^{*}$ and $2C^{0}=C+C^{*}$, the latter relation becomes:

(20) $$\begin{array}{cc} 2\tau (p)= & 2{\left(m+1\right)}^{2}∥H^{0}{∥}^{2}-\frac{{\left(m+1\right)}^{2}}{2}{∥H∥}^{2}-\frac{{\left(m+1\right)}^{2}}{2}{∥H^{*}∥}^{2}\\ & -2\left(m+1\right)C^{0}+\frac{m+1}{2}\left(C+C^{*}\right)+\frac{3(c+1)}{4}{∥P∥}^{2}\\ & +\frac{m}{4}\left[c\left(m-1\right)-3m-5\right]. \end{array}$$

Let P be the quadratic polynomial in the components of the second fundamental form defined by

(21) $$\begin{array}{ccc} P & = & rC^{0}+a\left(r\right)\;C^{0}\left(L\right)+\frac{m+1}{2}\left(C+C^{*}\right)-\frac{{\left(m+1\right)}^{2}}{2}{(∥H∥}^{2}+∥H^{*}{∥}^{2})\\ & & -2\tau \left(p\right)+\frac{3(c+1)}{4}{∥P∥}^{2}+\frac{m}{4}\left[c\left(m-1\right)-3m-5\right], \end{array}$$

where *L* is a hyperplane of $T_{p}M$.

Suppose that the hyperplane *L* is spanned by the tangent vectors $e_{1},\ldots ,e_{m}$, avoiding loss of generality. Then, from Equations (20) and (21), we derive that P has the expression

(22) $$\begin{array}{c} P=\sum_{\alpha =m+2}^{2n+1}\left[\frac{2m+r+2}{m+1}\sum_{i,j=1}^{m+1}{\left(h_{ij}^{0\alpha }\right)}^{2}+a\left(r\right)\frac{1}{m}\sum_{i,j=1}^{m}{\left(h_{ij}^{0\alpha }\right)}^{2}-2{\left(\sum_{i=1}^{m+1}h_{ii}^{0\alpha }\right)}^{2}\right]. \end{array}$$

Moreover, from Equation (22), we get:

$$\begin{array}{ccc} P & = & \sum_{\alpha =m+2}^{2n+1}\{\left[\frac{2(2m+r+2)}{m+1}+\frac{2a(r)}{m}\right]\sum_{1\leq i<j\leq m}{\left(h_{ij}^{0\alpha }\right)}^{2}+\frac{2(2m+r+2)}{m+1}\sum_{i=1}^{m}{\left(h_{i\;m+1}^{0\alpha }\right)}^{2}\\ & & +\left(\frac{2m+r+2}{m+1}+\frac{a(r)}{m}-2\right)\sum_{i=1}^{m}{\left(h_{ii}^{0\alpha }\right)}^{2}\\ & & -4\sum_{1\leq i<j\leq m+1}h_{ii}^{0\alpha }h_{jj}^{0\alpha }+\left(\frac{2m+r+2}{m+1}-2\right){\left(h_{m+1\;m+1}^{0\alpha }\right)}^{2}\}\\ & \geq & \sum_{\alpha =m+2}^{2n+1}\left[\frac{rm+a(r)(m+1)}{m(m+1)}\sum_{i=1}^{m}{\left(h_{ii}^{0\alpha }\right)}^{2}+\left(\frac{r}{m+1}\right){\left(h_{m+1\;m+1}^{0\alpha }\right)}^{2}-4\sum_{1\leq i<j\leq m+1}h_{ii}^{0\alpha }h_{jj}^{0\alpha }\right]. \end{array}$$

Let $f_{\alpha }$ be a quadratic form defined for any α∈{m+2,…,2n+1} by $f_{\alpha }:R^{m+1}\to R,$

$$\begin{array}{ccc} f_{\alpha }\left(h_{11}^{0\alpha },h_{22}^{0\alpha },\ldots ,h_{m+1\;m+1}^{0\alpha }\right) & = & \sum_{i=1}^{m}\frac{rm+a(r)(m+1)}{m(m+1)}{\left(h_{ii}^{0\alpha }\right)}^{2}\\ & & +\frac{r}{m+1}{\left(h_{m+1\;m+1}^{0\alpha }\right)}^{2}-4\sum_{1\leq i<j\leq m+1}h_{ii}^{0\alpha }h_{jj}^{0\alpha }. \end{array}$$

We study the constrained extremum problem

$$\min f_{\alpha }$$

with the constraint

$$Q:h_{11}^{0\alpha }+h_{22}^{0\alpha }+\ldots +h_{m+1\;m+1}^{0\alpha }=k^{\alpha },$$

where $k^{\alpha }$ is a real constant.

The first order partial derivatives system is:

$$\left\{\begin{array}{c} \frac{\partial f_{\alpha }}{\partial h_{ii}^{0\alpha }}=2\frac{rm+a(r)(m+1)}{m(m+1)}h_{ii}^{0\alpha }-4\left(\sum_{k=1}^{m+1}h_{kk}^{0\alpha }-h_{ii}^{0\alpha }\right)=0\\ \frac{\partial f_{\alpha }}{\partial h_{m+1\;m+1}^{0\alpha }}=\frac{2r}{m+1}h_{m+1\;m+1}^{0\alpha }-4\sum_{k=1}^{m}h_{kk}^{0\alpha }=0, \end{array}\right.$$

for every i∈{1,…,m}, α∈{m+2,…,2n+1}.

The system solution, satisfying the constraint *Q*, is the critical point with the expression:

$$h_{ii}^{0\alpha }=\frac{2m(m+1)}{2m^{2}+\left[r+a\left(r\right)+2\right]m+a\left(r\right)}k^{\alpha },$$

$$h_{m+1\;m+1}^{0\alpha }=\frac{2(m+1)}{2m+r+2}k^{\alpha },$$

for any i∈{1,…,m}, α∈{m+2,…,2n+1}.

Let *p* be an arbitrary point of *Q*, p∈ Q. We consider that the 2-form $A:T_{p}Q\times T_{p}Q\to R$ given by:

$$A\left(X,Y\right)=\mathrm{Hess}\left(f_{\alpha }\right)\left(X,Y\right)+\langle h^{\prime }\left(X,Y\right),\left(\mathrm{grad}f_{\alpha }\right)\left(p\right)\rangle ,$$

where $h^{\prime }$ is the second fundamental form of *Q* in $R^{m+1}$ and 〈·,·〉 is the standard inner product on $R^{m+1}$.

The Hessian matrix of $f_{\alpha }$ is as follows:

$$\mathrm{Hess}\left(f_{\alpha }\right)=\left(\begin{array}{ccccc} b & -4 & \cdots & -4 & -4\\ -4 & b & \cdots & -4 & -4\\ \vdots & \vdots & \ddots & \vdots & \vdots \\ -4 & -4 & \cdots & b & -4\\ -4 & -4 & \cdots & -4 & \frac{2r}{m+1} \end{array}\right),$$

where *b* is a real constant, namely

$$b=2\frac{rm+a\left(r\right)\left(m+1\right)+2m^{2}+2m}{m(m+1)}.$$

As the hyperplane *Q* is totally geodesic in $R^{m+1}$, considering a vector field $X\in T_{p}Q$, that is satisfying the condition $\sum_{i=1}^{m+1}X_{i}=0$, we get

$$\begin{array}{cc} A(X,X) & =b\sum_{i=1}^{m}X_{i}^{2}+\frac{2r}{m+1}X_{m+1}^{2}-8\sum_{i,j=1(i\neq j)}^{m+1}X_{i}X_{j}\\ & =b\sum_{i=1}^{m}X_{i}^{2}+\frac{2r}{m+1}X_{m+1}^{2}+4{\left(\sum_{i=1}^{m+1}X_{i}\right)}^{2}-8\sum_{i,j=1(i\neq j)}^{m+1}X_{i}X_{j}\\ & =b\sum_{i=1}^{m}X_{i}^{2}+\frac{2r}{m+1}X_{m+1}^{2}+4\sum_{i=1}^{m+1}X_{i}^{2}\\ & \geq 0. \end{array}$$

However, according to the Remark 2, the critical point $\left(h_{11}^{0\alpha },\ldots ,h_{m+1\;m+1}^{0\alpha }\right)$ is the only optimal solution, i.e., the global minimum point of problem. In addition, $f_{\alpha }\left(h_{11}^{0\alpha },\ldots ,h_{m+1\;m+1}^{0\alpha }\right)=0$. Thus, we obtain P≥0 and this implies

$$\begin{array}{ccc} 2\tau & \leq & rC^{0}+a\left(r\right)\;C^{0}\left(L\right)+\frac{m+1}{2}\left(C+C^{*}\right)-\frac{{\left(m+1\right)}^{2}}{2}{(∥H∥}^{2}+∥H^{*}{∥}^{2})\\ & & +\frac{3(c+1)}{4}{∥P∥}^{2}+\frac{m}{4}\left[c\left(m-1\right)-3m-5\right], \end{array}$$

for every tangent hyperplane *L* of $T_{p}M$. Consequently, we get immediately both inequalities Equations (15) and (16) from the above relation, taking infimum and supremum respectively, over all tangent hyperplanes *L* of $T_{p}M$.

Next, we investigate the equality cases of the inequalities Equations (15) and (16). First of all, we determine the critical points

$$h^{c}=\left(h_{11}^{0\;m+2},h_{12}^{0\;m+2},\ldots ,h_{m+1\;m+1}^{0\;m+2},\ldots ,h_{11}^{0\;2n+1},\ldots ,h_{m+1\;m+1}^{0\;2n+1}\right)$$

of P as the solutions of following system of linear homogeneous equations:

$$\left\{\begin{array}{c} \frac{\partial P}{\partial h_{ii}^{0\alpha }}=2\left[\frac{2m+r+2}{m+1}+\frac{a(r)}{m}-2\right]h_{ii}^{0\alpha }-4\sum_{k\neq i,k=1}^{m+1}h_{kk}^{0\alpha }=0,\\ \frac{\partial P}{\partial h_{m+1\;m+1}^{0\alpha }}=2\frac{r}{m+1}h_{m+1\;m+1}^{0\alpha }-4\sum_{k=1}^{m}h_{kk}^{0\alpha }=0,\\ \frac{\partial P}{\partial h_{ij}^{0\alpha }}=4\left[\frac{2m+r+2}{m+1}+\frac{a(r)}{m}\right]h_{ij}^{0\alpha }=0,\;\;i\neq j,\\ \frac{\partial P}{\partial h_{i\;m+1}^{0\alpha }}=\frac{4(2m+r+2)}{m+1}h_{i\;m+1}^{0\alpha }=0. \end{array}\right.$$

We achieve $h_{ij}^{0\alpha }=0$, with i,j∈{1,…,m+1} and α∈{m+2,…,2n+1}. Because P≥0 and $P(h^{c})=0$, then the critical point $h^{c}$ is a minimum point of P. Therefore, the equality cases hold in both inequalities Equations (15) and (16) if and only if $h_{ij}^{\alpha }=-h_{ij}^{*\alpha },$ for i,j∈{1,…,m+1}, α∈{m+2,…,2n+1}, and the conclusion is now clear.  ☐

**Remark** **3.** *Equation* (17) *signifies that the submanifold M is totally geodesic with respect to the connection ${\bar{\nabla }}^{0}$. Hence, the equality case at all points in both inequalities (7) and (8) characterizes the totally geodesic submanifolds with respect to the Levi–Civita connection.*

**Corollary** **1.** *Let M be an (m+1)-dimensional statistical submanifold of a (2n+1)-dimensional Kenmotsu statistical manifold ($\bar{M},\bar{\nabla },\bar{g},\phi ,\xi$) of constant ϕ-sectional curvature c. Then:*

*(i)*  *The normalized δ-Casorati curvatures $\delta_{C}\left(m\right)$ and $\delta_{C}^{*}\left(m\right)$ satisfy*
  (23) $$\begin{array}{ccc} \rho & \leq & \delta_{C}^{0}\left(m\right)+\frac{1}{m}C^{0}-\frac{2(m+1)}{m}{∥H^{0}∥}^{2}\\ & + & \frac{m+1}{m}\bar{g}\left(H,H^{*}\right)+\frac{3(c+1)}{4m(m+1)}{∥P∥}^{2}+\frac{c(m-1)-3m-5}{4(m+1)}, \end{array}$$
  *where $2\delta_{C}^{0}\left(m\right)=\delta_{C}\left(m\right)+\delta_{C}^{*}\left(m\right)$ and $2C^{0}=C+C^{*}$.*
*(ii)*  *The normalized δ-Casorati curvatures ${\hat{\delta }}_{C}\left(m\right)$ and ${\hat{\delta }}_{C}^{*}\left(m\right)$ satisfy*
  (24) $$\begin{array}{ccc} \rho & \leq & {\hat{\delta }}_{C}^{0}\left(m\right)+\frac{1}{m}C^{0}-\frac{2(m+1)}{m}{∥H^{0}∥}^{2}\\ & + & \frac{m+1}{m}\bar{g}\left(H,H^{*}\right)+\frac{3(c+1)}{4m(m+1)}{∥P∥}^{2}+\frac{c(m-1)-3m-5}{4(m+1)}, \end{array}$$
  *where $2{\hat{\delta }}_{C}^{0}\left(m\right)={\hat{\delta }}_{C}\left(m\right)+{\hat{\delta }}_{C}^{*}\left(m\right)$.*

*Moreover, the equality cases of Equations* (23) *and* (24) *hold identically at all points if and only if the imbedding curvature tensors h and $h^{*}$ of the submanifold associated with the dual connections $\bar{\nabla }$ and ${\bar{\nabla }}^{*}$ satisfy Equation* (17)*, i.e., M is a totally geodesic submanifold with respect to the Levi–Civita connection.*

**Proof.**  The conclusion follows immediately from Theorem 2, taking account of Equations (8)–(10).  ☐

**Remark** **4.** *Corollary 1 is the statistical counterpart of the Theorem 1 from* [20].

## 4. An Example and Concluding Remarks

**Remark** **5.** *Any Kenmotsu manifold can be obtained locally as follows (see (*[45]*, Proposition 3.2)). Let $\left(M_{0},g_{0},J\right)$ be an almost Hermitian manifold. Set $\bar{M}=M_{0}\times R,$$\bar{g}=e^{2t}g_{0}+{\left(dt\right)}^{2},$$\xi =\frac{\partial }{\partial t}\in \Gamma \left(T\bar{M}\right),$ and define $\phi \in \Gamma (T{\bar{M}}^{\left(1,1\right)})$ by ϕU=JU, for any $U\in \Gamma (T\bar{M})$ and ϕξ=0. Then:*

*1.*  The triple ($\bar{g},\phi ,\xi$) is an almost contact metric structure on $\bar{M}$.
*2.*  The pair $\left(g_{0},J\right)$ is a Kähler structure on $M_{0}$ if and only if ($\bar{g},\phi ,\xi$) is a Kenmotsu structure on $\bar{M}$.

**Remark** **6.** *Let $\left(M_{0},g_{0},J\right)$ be a Kähler manifold and $\left(\bar{M}=M_{0}\times R,\bar{g},\phi ,\xi \right)$ be the Kenmotsu manifold as in Remark 5. Let $\left(\bar{\nabla }=\nabla^{\bar{g}}+\bar{K},\bar{g}\right)$ be a statistical structure on $\bar{M}$. Define $\Lambda \in \Gamma (T{\bar{M}}^{\left(0,2\right)}⊗TM_{0})$, $\lambda \in \Gamma (T{\bar{M}}^{\left(0,2\right)})$ and $K\in \Gamma (TM_{0})$ as in* ([45]*, Theorem 3.8*)*. Then, it follows that $\left(\bar{\nabla }=\nabla^{\bar{g}}+\bar{K},\bar{g}\right)$ is a Kenmotsu statistical structure on $\bar{M}$ if and only if $\left(\nabla =\nabla^{g_{0}}+K,g_{0},J\right)$ is an holomorphic statistical structure on $M_{0}$, and the formulas Λ(X,ξ)=0, λ(X,V)=0 hold for $X\in \Gamma (T\bar{M})$ and $V\in \Gamma (TM_{0})$. Moreover, if the Kenmotsu statistical manifold $\left(\bar{M}=M_{0}\times R,\bar{\nabla }=\nabla^{g}+\bar{K},\bar{g},\phi ,\xi \right)$ is of constant ϕ-sectional curvature c, then c=−1 and $\left(M_{0},\nabla =\nabla^{g_{0}}+K,g_{0},J\right)$ is of constant holomorphic sectional curvature 0 (see (*[45]*, Proposition 3.9)).*

*Hence, from Theorem 2 and Corollary 1, we derive the following results.*

**Theorem** **3.** *Let $\left(M_{0},\nabla =\nabla^{g_{0}}+K,g_{0},J\right)$ be a holomorphic statistical manifold of constant holomorphic sectional curvature 0 and $\left(\bar{M}=M_{0}\times R,\bar{\nabla }={\bar{\nabla }}^{\bar{g}}+\bar{K},\bar{g},\phi ,\xi \right)$ be the Kenmotsu statistical manifold of constant ϕ-sectional curvature constructed as in Remark 6. If M is an (m+1)-dimensional statistical submanifold of $\bar{M}$, then:*

*(i)*  *For any real number r such that 0<r<m(m+1), the generalized normalized δ-Casorati curvatures $\delta_{C}\left(r;m\right)$ and $\delta_{C^{*}}\left(r;m\right)$ satisfy*
  (25) $$\begin{array}{ccc} 2\tau & \leq & \delta_{C}^{0}\left(r;m\right)+\left(m+1\right)C^{0}-2{\left(m+1\right)}^{2}{∥H^{0}∥}^{2}\\ & + & {\left(m+1\right)}^{2}\bar{g}\left(H,H^{*}\right)-m\left(m+1\right), \end{array}$$
  *where $2\delta_{C}^{0}\left(r,m\right)=\delta_{C}\left(r;m\right)+\delta_{C}^{*}\left(r;m\right)$ and $2C^{0}=C+C^{*}$.*
*(ii)*  *For any real number r such that r>m(m+1), the generalized normalized δ-Casorati curvatures ${\hat{\delta }}_{C}\left(r;m\right)$ and ${\hat{\delta }}_{C}^{*}\left(r;m\right)$ satisfy*
  (26) $$\begin{array}{ccc} 2\tau & \leq & {\hat{\delta }}_{C}^{0}\left(r;m\right)+\left(m+1\right)C^{0}-2{\left(m+1\right)}^{2}{∥H^{0}∥}^{2}\\ & + & {\left(m+1\right)}^{2}\bar{g}\left(H,H^{*}\right)-m\left(m+1\right), \end{array}$$
  *where $2{\hat{\delta }}_{C}^{0}={\hat{\delta }}_{C}\left(r;m\right)+{\hat{\delta }}_{C}^{*}\left(r;m\right)$.*

*Moreover, the equality cases of Equations* (25) *and* (26) *hold identically at all points if and only if the imbedding curvature tensors h and $h^{*}$ of the submanifold associated to the dual connections $\bar{\nabla }$ and ${\bar{\nabla }}^{*}$ satisfy Equation* (17)*, i.e., M is a totally geodesic submanifold with respect to the Levi–Civita connection.*

**Corollary** **2.** *Let $\left(M_{0},\nabla =\nabla^{g_{0}}+K,g_{0},J\right)$ be an holomorphic statistical manifold of constant holomorphic sectional curvature 0 and $\left(\bar{M}=M_{0}\times R,\bar{\nabla }={\bar{\nabla }}^{\bar{g}}+\bar{K},\bar{g},\phi ,\xi \right)$ be the Kenmotsu statistical manifold of constant ϕ-sectional curvature constructed as in Remark 6. If M is an (m+1)-dimensional statistical submanifold of $\bar{M}$, then:*

*(i)*  *The normalized δ-Casorati curvatures $\delta_{C}\left(m\right)$ and $\delta_{C}^{*}\left(m\right)$ satisfy*
  (27) $$\begin{array}{c} \rho \leq \delta_{C}^{0}\left(m\right)+\frac{1}{m}C^{0}-\frac{2(m+1)}{m}{∥H^{0}∥}^{2}+\frac{m+1}{m}\bar{g}\left(H,H^{*}\right)-1 \end{array}$$
  *where $2\delta_{C}^{0}\left(m\right)=\delta_{C}\left(m\right)+\delta_{C}^{*}\left(m\right)$ and $2C^{0}=C+C^{*}$.*
*(ii)*  *The normalized δ-Casorati curvature ${\hat{\delta }}_{C}\left(m\right)$ and ${\hat{\delta }}_{C}^{*}\left(m\right)$ satisfy*
  (28) $$\begin{array}{c} \rho \leq {\hat{\delta }}_{C}^{0}\left(m\right)+\frac{1}{m}C^{0}-\frac{2(m+1)}{m}{∥H^{0}∥}^{2}+\frac{m+1}{m}\bar{g}\left(H,H^{*}\right)-1, \end{array}$$
  *where $2{\hat{\delta }}_{C}^{0}\left(m\right)={\hat{\delta }}_{C}\left(m\right)+{\hat{\delta }}_{C}^{*}\left(m\right)$.*

*Moreover, the equality cases of Equations* (27) *and* (28) *hold identically at all points if and only if the imbedding curvature tensors h and $h^{*}$ of the submanifold associated with the dual connections $\bar{\nabla }$ and ${\bar{\nabla }}^{*}$ satisfy Equation* (17)*, i.e., M is a totally geodesic submanifold with respect to the Levi–Civita connection.*

**Example** **1.** *We consider the Kenmotsu manifold $\left(H^{2n+1},\bar{g},\phi ,\xi \right)$ from* ([45]*, Example 3.3*)*. We recall that*

$$H^{2n+1}=\left\{\left(x^{1},\ldots ,x^{n},y^{1},\ldots ,y^{n},z\right)\in R^{2n+1}|z>0\right\}$$

*and the structure tensors $\left(\bar{g},\phi ,\xi \right)$ are given by*

$$\bar{g}=z^{-2}\left\{{\left(dx^{1}\right)}^{2}+\ldots +{\left(dx^{n}\right)}^{2}+{\left(dy^{1}\right)}^{2}+\ldots +{\left(dy^{n}\right)}^{2}+{\left(dz\right)}^{2}\right\},$$

$$\phi \frac{\partial }{\partial x^{\alpha }}=\frac{\partial }{\partial y^{\alpha }},\;\phi \frac{\partial }{\partial y^{\alpha }}=-\frac{\partial }{\partial x^{\alpha }},\;\phi \frac{\partial }{\partial z}=0$$

*and*

$$\xi =-z\frac{\partial }{\partial z}.$$

*In the following, we set*

$$\bar{K}\left(X,Y\right)=\nu \;\eta \left(X\right)\;\eta \left(Y\right)\;\xi ,$$

*for any $X,Y\in \Gamma (TH^{2n+1})$ and $\nu \in C^{\infty }\left(H^{2n+1}\right)$, where η is the 1-form on $H^{2n+1}$ given by $\eta \left(\cdot \right)=\bar{g}\left(\cdot ,\xi \right)$. Then, it follows that $\left(H^{2n+1},\bar{\nabla }={\bar{\nabla }}^{\bar{g}}+\bar{K},\bar{g},\phi ,\xi \right)$ is a Kenmotsu statistical manifold with constant ϕ-sectional curvature c=−1(see (*[45]*, Example 3.10)).*

*Next, let M be any (m+1)-dimensional submanifold of the Kenmotsu statistical manifold $H^{2n+1}$. Then, the inequalities Equations* (25) *and* (26) *are satisfied. In particular, the statistical submanifold $M=H^{2p+1}$ of $H^{2n+1}$, with 0<p<n, attains equality in both inequalities Equations* (25) *and* (26) *because M is a totally geodesic submanifold of $H^{2n+1}$ with respect to the Levi–Civita connection.*

## 5. Conclusions

It is well known that many applications of Amari’s dual geometries involve one or more submanifolds imbedded in a manifold [33]. In particular, it follows that it is of great interest to find simple relationships between various invariants of the submanifolds and manifolds. In this work, using the fundamental equations for statistical submanifolds, we established such relationships between some basic extrinsic and intrinsic invariants of statistical submanifolds in Kenmotsu statistical manifolds of constant ϕ-sectional curvature. The results stated here motivate further studies to obtain similar relationships for many kinds of invariants of similar nature, for statistical submanifolds in several ambient spaces, like holomorphic statistical manifolds [46], Sasakian statistical manifolds [38] and cosymplectic statistical manifold [49].
